# Urinary neopterin reflects immunological variation associated with age, helminth parasitism, and the microbiome in a wild primate

**DOI:** 10.1038/s41598-022-25298-9

**Published:** 2022-12-09

**Authors:** India A. Schneider-Crease, Jacob A. Feder, Alice Baniel, Colleen McCann, Abebaw Azanaw Haile, Belayneh Abebe, Lauren Fitzgerald, Megan A. Gomery, Ruth A. Simberloff, Zack L. Petrie, Sarah Gabriel, Pierre Dorny, Peter J. Fashing, Nga Nguyen, Thore J. Bergman, Jacinta C. Beehner, Noah Snyder-Mackler, Amy Lu

**Affiliations:** 1 School of Human Evolution and Social Change, Arizona State University, Tempe, AZ USA; 2 Center for Evolution and Medicine, Arizona State University, Tempe, AZ USA; 3 Interdepartmental Doctoral Program in Anthropological Sciences, Stony Brook University, Stony Brook, NY USA; 4 School of Life Sciences, Arizona State University, Tempe, AZ USA; 5 Department of Mammals, Bronx Zoo, Wildlife Conservation Society, New York, NY USA; 6 New York Consortium in Evolutionary Primatology, New York, NY USA; 7 Ethiopian Wildlife Conservation Authority, Addis Ababa, Ethiopia; 8 African Wildlife Foundation, Simien Mountains Landscape Conservation and Management Project, Debark, Ethiopia; 9 Department of Biology, Miami University, Oxford, OH USA; 10 Simien Mountains Gelada Research Project, Debark, Ethiopia; 11 Department of Ecology and Evolutionary Biology, University of Tennessee, Knoxville, TN USA; 12 Guassa Gelada Research Project, Guassa, Ethiopia; 13 Department of Translational Physiology, Infectiology and Public Health, Faculty of Veterinary Medicine, Ghent University, Ghent, Belgium; 14 Department of Biomedical Sciences, Institute for Tropical Medicine, Antwerp, Belgium; 15 Department of Anthropology, California State University Fullerton, Fullerton, CA USA; 16 Centre for Ecological and Evolutionary Synthesis, Department of Biosciences, University of Oslo, Oslo, Norway; 17 Department of Ecology and Evolution, University of Michigan, Ann Arbor, MI USA; 18 Department of Psychology, University of Michigan, Ann Arbor, MI USA; 19 Department of Anthropology, University of Michigan, Ann Arbor, MI USA; 20 Department of Anthropology, Stony Brook University, Stony Brook, NY USA

**Keywords:** Ecology, Zoology, Diseases

## Abstract

Neopterin, a product of activated white blood cells, is a marker of nonspecific inflammation that can capture variation in immune investment or disease-related immune activity and can be collected noninvasively in urine. Mounting studies in wildlife point to lifetime patterns in neopterin related to immune development, aging, and certain diseases, but rarely are studies able to assess whether neopterin can capture multiple concurrent dimensions of health and disease in a single system. We assessed the relationship between urinary neopterin stored on filter paper and multiple metrics of health and disease in wild geladas (*Theropithecus gelada*), primates endemic to the Ethiopian highlands. We tested whether neopterin captures age-related variation in inflammation arising from developing immunity in infancy and chronic inflammation in old age, inflammation related to intramuscular tapeworm infection, helminth-induced anti-inflammatory immunomodulation, and perturbations in the gastrointestinal microbiome. We found that neopterin had a U-shaped relationship with age, no association with larval tapeworm infection, a negative relationship with metrics related to gastrointestinal helminth infection, and a negative relationship with microbial diversity. Together with growing research on neopterin and specific diseases, our results demonstrate that urinary neopterin can be a powerful tool for assessing multiple dimensions of health and disease in wildlife.

## Introduction

Ecological and evolutionary studies of health and disease in natural settings require the implementation of noninvasive diagnostics. These noninvasive diagnostics can facilitate longitudinal monitoring of infectious diseases in wildlife, allow for the assessment of changes in health connected to climate change or other human-mediated environmental disruptions, and permit the study of physiological phenomena with minimal interruption to the study subjects. Indeed, a number of noninvasive tools have been developed over the past few decades, most of which focus on identification of a particular pathogen or community of pathogens through fecal- or urine-based analysis^1–5^ or on metabolites related to the endocrine response^6–8^. More recent research has begun to develop noninvasive markers of the immune response, including those that can be readily measured in urine such as C-reactive protein, soluble urokinase plasminogen activator receptor (suPAR), and neopterin^9–11^. Among these urinary biomarkers, neopterin has been increasingly implemented in studies of wildlife health and disease^12–19^.

Neopterin is a pteridine produced by interferon-gamma (IFN-y)—activated macrophages and monocytes during the T-helper cell-mediated immune response, and is therefore expected to closely mirror systemic inflammation. Elevated neopterin levels, which are detectable at biologically meaningful and largely concordant levels in blood and urine^20^, can reflect changes in generalized immune investment across the lifespan. High neopterin levels in adults may capture the chronic inflammation that accompanies chronological aging^21^, while high neopterin levels in infants may capture the T-cell helper (Th) 2-polarization of the developing immune system^22^. Studies that sampled humans, semi-wild mandrills, and captive and wild capuchins across the lifespan found a U-shaped relationship between neopterin and age, with the highest neopterin in the youngest and oldest individuals^19,23,24^. Concordantly, studies that sampled only adults found the highest neopterin in older individuals^16,18^ and those that sampled only non-adults found the highest neopterin in younger individuals^12,14^.

Elevated neopterin levels may also reflect immune activity related to autoimmune, noncommunicable, or infectious diseases. A number of autoimmune diseases, heart and kidney failure, and coronary artery disease are all associated with significant increases in serum neopterin levels^25–28^, as are infectious diseases that recruit the inflammatory immune response, including *Plasmodium falciparum* malaria, tuberculosis, SIV, and hepatitis A, B, and C^20,29,30^. Neopterin can also be a useful marker of infection progress, with levels tracking the severity of clinical symptoms in dengue fever^31^. Across primate studies, urinary neopterin levels have been associated with respiratory disease symptoms in captive bonobos^15^, deaths involving respiratory disease in wild chimpanzees^32^, SIV viremia in captive rhesus and long-tailed macaques^11^, and *Plasmodium gonderi* parasitemia in wild mandrills^19^. Neopterin might also be expected to capture other elements of health and disease—for example, lower neopterin levels could reflect the regulatory and anti-inflammatory properties of chronic helminth infections^33–35^ while higher neopterin levels might capture tissue damage from migratory or intramuscular stages of parasite infections (e.g., hookworm, larval tapeworms^36,37^) or disruptions in microbiome communities that play a critical role in immune balance and regulation^38^. Because urinary neopterin is a recent contribution to the noninvasive diagnostics toolkit, few studies have been able to assess the relationship between neopterin and multiple metrics of health and disease in a single system.

As a contribution towards defining appropriate uses of urinary neopterin as a biomarker of inflammation or disease in wildlife, we investigated the relationship between urinary neopterin, demographic variables, and metrics of health, disease, and parasite infection in wild populations of geladas (*Theropithecus gelada*), a cercopithecine primate endemic to the Ethiopian highlands. Specifically, we assess whether neopterin captures age-related variation in baseline inflammation, expecting to observe the highest neopterin in the youngest and oldest individuals. We also evaluate whether urinary neopterin captures (1) inflammation associated with the intramuscular larval tapeworm *Taenia serialis*, which infects wild geladas and causes protuberant cysts that substantially increase mortality^39,40^, (2) regulatory and anti-inflammatory effects of gastrointestinal parasite infection^41^, and (3) characteristics of the gastrointestinal microbiota that reflect dysregulation (i.e., low alpha diversity, abundance of pathogenic bacteria).

## Materials and methods

We sampled across age-sex classes from the only two populations of wild geladas under long-term study in the Ethiopian highlands. We collected urine samples to assess urinary neopterin and *T. serialis* infection status between 2018 and 2020 from one gelada population in the Simien Mountains National Park (SMNP; 13.183’N, 38.0667’E) under long-term study by the Simien Mountains Gelada Research Project and from another population between 2018–2019 in the Guassa Community Conservation Area (GCCA; N 10°15′–10°27′; E 39°45′–39°49′) under long-term study by the Guassa Gelada Research Project. We additionally collected fecal samples for gastrointestinal parasite and microbiome characterization from the SMNP population (see Table 1 for all analyses and sample sizes). Geladas at both sites are individually recognizable by the respective field teams based on suites of morphological characteristics.

**Table 1:** Analyses of urinary neopterin predictors in wild gelada populations in the Simien Mountains National Park (SMNP) and the Guassa Community Conservation Area (GCCA).

Analysis	Predictors	N (SMNP)	N (GCCA)
1. Demographic, climatic, and technical factors	Age, sex, rainfall, min temperature, time of day, storage time	588	NA
2. *Taenia serialis*	Age, time of day, storage time, *T. serialis* infection	128	45
3. Gastrointestinal helminths	Age, time of day, storage time, helminth ASV richness over 1 year prior to sampling	140	NA
4. Microbiome 4.1 Alpha diversity 4.2 Beta diversity	Age, time of day, storage time, microbiome ASV richness, Shannon’s diversity index, Faith’s phylogenetic diversity index Age, time of day, storage time, Bray–Curtis diversity, weighted and unweighted UniFrac distances	229	NA

### Urine sample collection

We collected urine samples for urinary neopterin from SMNP geladas (n = 588 samples from 110 individuals, age range based on known or estimated dates of birth = 0.4–21.0 years, mean age = 5.6 years, standard deviation = 4 years) between February 2018- March 2020 and from GCCA geladas (n = 45 samples from 26 individuals, age range based on known or estimated dates of birth = 1.9–15.8 years; mean ± SD = 9 ± 3.5 years) between September 2018- January 2019. We estimated dates of birth for adult females and males when known dates of birth were not available. We assigned estimated dates of birth for adult females by back-calculating from observed sexual maturation dates using the average age at maturation in geladas (4.65 years)^42^. We assigned estimated dates of birth for adult males by subtracting the median age of validated maturational categories (based on suites of physical traits^43^) from the date of first sample collection. Samples were stored at room temperature until analysis, which ranged from 101–985 days (mean ± SD = 534 ± 304 days). In addition to samples collected from wild geladas, we collected urine samples from geladas at the Bronx Zoo (n = 14 samples from seven adults) in 2018 to assess the accuracy and integrity of samples on filter paper and compared to samples that were immediately frozen. Urine from both wild populations was collected from the ground immediately following urination with filter papers (Whatman Qualitative Filter Papers, Grade 4, 11.0 cm), which were stored in labeled Whirl–Pak © bags with ~ 1 g of silica desiccant, and urine from geladas housed in zoos was pipetted directly into microcentrifuge tubes and then pooled.

### Neopterin assay validation and urine sample analysis

Using a commercial neopterin ELISA kit (IBL International GmbH, Hamburg, Germany, RE59321), we first assessed parallelism between the kit-derived standard curve and two serial dilutions: one from a urine pool from the zoo gelada population and one from a urine pool from reconstituted filter paper samples from the SMNP population (Supplementary Fig. 1 (Fig. S1)). We detected no difference in slope between the standard curve and the serial dilutions derived from frozen samples (ANCOVA: *R* = 0.99, *F*(1,7) = 0.72, *p* = 0.43) and from filter paper samples (ANCOVA: *R* = 0.99, *F*(1,6) = 5.09, *p* = 0.07; Fig. S1). Second, we assessed accuracy by spiking kit standards into a low-neopterin sample derived from filter paper samples collected from the wild gelada population and assaying in quadruplicate. Average recovery of the spiked sample was 97.8 ± 5.9%.

We used zoo samples to assess the stability of neopterin in filter paper samples compared to frozen wet samples across three time intervals (0, 4, and 8 months following collection). Because neopterin values are typically standardized to urinary creatinine to control for urine concentration^44^, we also measured urinary creatinine with a commercial creatinine kit (Arbor Assays, Michigan, USA, #K002-H). Samples were correlated across storage media (frozen vs. filter paper), across time points (0, 4, and 8 months), and across each measurement of interest (urinary neopterin, creatinine, and neopterin/creatine index; Fig. S2).

Urine samples collected from wild geladas were reconstituted in deionized water using a 1:12 dilution so that samples fell in the mid-range of the standard curve (i.e., between 20 and 80% binding). Samples were then assayed for urinary neopterin and urinary creatinine with the kits described above. Neopterin intra-assay CVs were 13.3% and 12.2% for low (1.57 ng/mL) and high (4.90 ng/mL) kit controls, respectively, while inter-assay CVs were 14.7% and 11.2% for low and high kit controls (n = 27), respectively.

### Demographic, environmental, and technical predictors of neopterin

To identify the broad demographic, environmental, and technical predictors of urinary neopterin, we modeled the effects of age (linear and quadratic), sex, and climatic variables on log-transformed neopterin index concentrations using a linear mixed model (LMM) in SMNP geladas (n = 588 urine samples from 110 individuals, 1–23 samples per individual). We elected to focus on samples from a single site to reduce any potential noise arising from cross-site comparisons and because GCCA samples only spanned several months in a single year, where SMNP samples spanned seasons over two years. We included variables based on our hypotheses and to capture potential sources of biological and technical variation. In the Ethiopian highlands, seasons are characterized by temperature and rainfall; thus, we included average minimum temperature in the 30 days (scaled by mean and standard deviation) before sample collection to capture physiological changes related to cold stress^45,46^ and total rainfall in the 90 days before sample collection (also scaled) as a proxy of food availability in this population^47^ that could modulate immunity^48,49^. We included scaled time of sample collection (as a proportion of the day) to control for potential diurnal variation and storage time to account for storage times that exceeded the length of storage experiments due to extenuating circumstances (i.e., the COVID-19 pandemic, geopolitical conflict at the study sites). Finally, we included individual ID and batch number for neopterin and creatinine as random effects. We assessed variance inflation factors to identify collinearity across our variables and found that all values were well below generally accepted thresholds (i.e., < 1.1). For all downstream analyses, we include only those predictors that were significantly associated with neopterin index in this primary model.

### Larval tapeworm infection and neopterin

Urine samples from the SMNP population (n = 128 samples from 60 individuals) and the GCCA population (n = 45 samples from 26 individuals) were analyzed for *T. serialis* infection from the same filter paper samples assayed for urinary neopterin and urinary creatinine as described above. We included samples from the GCCA population in this analysis because of their relatively higher prevalence of *T. serialis* infections^39,40^. We determined *T. serialis* infection with a monoclonal antibody-based enzyme-linked immunosorbent assay following an established protocol validated for use in gelada urine^1^ with minor adjustments (Fig. S3).

We modeled the effects of positive *T. serialis* diagnosis (i.e., *Taenia* antigen corrected for creatinine concentration, expressed in ng/mL) on log-transformed neopterin indices using an LMM, predicting that infection would be associated with increased neopterin. We included variables that had significant explanatory power in the primary neopterin analysis (i.e., scaled storage time, linear and quadratic estimated age, and time of collection) and sampling site (i.e., SMNP, GCCA) as fixed effects and included individual ID, neopterin and creatinine batch numbers, and *Taenia* assay number as random effects.

### Gastrointestinal helminth parasites and neopterin

Fecal samples from the SMNP gelada population collected from these individuals over the same time period were analyzed for gastrointestinal nematode parasites with high-throughput amplicon sequencing of the Internal Transcription Spacer 2 (ITS-2), which was previously validated for use in gelada fecal samples^2^. Our final dataset included a total of 15 amplicon sequence variants (ASVs) assigned at the genus level to either *Trichostrongylus* spp. or *Oesophagostomum* spp. with > 92% percent identity match. Given the low genus richness in geladas, we calculated the richness of ASVs (i.e., the number of unique ASVs) in each sample.

Urine samples were matched with all fecal samples from each individual over the year prior to urine sample collection to capture long-term patterns in chronic gastrointestinal parasite community structure and impact on inflammatory profiles, culminating in a dataset of 140 urine samples matched to 49 fecal samples from 32 individuals (age range: 0.6–20 years, 2.9 average fecal samples per neopterin sample; mean ± SD = 61.8 ± 45.3 days apart between samples, range = 0–147 days). We then assessed the relationship between gastrointestinal parasite ASV richness and log-transformed neopterin index using an LMM, predicting that higher richness would be associated with lower neopterin levels as a result of the helminth-induced anti-inflammatory immune phenotype. We included age, age^2^, sample collection time, and sample storage time as fixed effects, and individual ID and neopterin and creatinine plate numbers as random effects.

### Gastrointestinal microbiome composition and neopterin

Fecal samples from the SMNP gelada population (N = 1267) were analyzed for gut microbiome composition using 16S rRNA amplicon sequencing (Fig. S5). We matched neopterin samples with gut microbiome sample(s) collected from the same individual over the month prior to the neopterin sample (mean ± SD = 13.4 ± 10.0 days apart between samples, range = 0–30 days) to capture short term effects of microbial characteristics on inflammatory profiles. The resulting dataset included 229 neopterin-microbiome matched datapoints (including 229 neopterin samples and 168 microbiome samples) from 55 individuals (age range: 0.5 to 21.0 years). For the 24 neopterin samples that had two associated microbiome samples within the prior month window, we averaged microbial metrics from the two samples.

We modeled the effect of age-adjusted alpha diversity (i.e., within-sample microbial diversity measured by the observed ASV richness, Shannon index and Faith’s phylogenetic diversity and corrected for the age of individuals; Fig. S5) on the log-transformed neopterin index using LMMs, expecting that lower microbial diversity would be associated with higher neopterin levels in the following month. We fit one model for each of the three age-adjusted alpha diversity metrics and included age (linear and quadratic), sample collection time, and sample storage time as fixed effects, with individual ID and neopterin and creatinine plate numbers as random effects.

We also tested whether neopterin indices were associated with differences in overall microbial composition by modeling the relationship between neopterin and microbial community features of beta diversity (i.e., between-sample dissimilarity in microbial community). Three complementary metrics of beta diversity (Bray–Curtis, unweighted and weighted UniFrac distances; Fig. S5) were calculated between each pair of fecal samples. We then reduced the dimensionality using Principal Coordinate Analysis (PCoA) and extracted sample coordinates from the first five Principal Coordinates (PCs, Fig. S5; Fig. S6). We fit one model for each of the three beta diversity metrics and included the corresponding first five beta diversity PCs as well as the same control predictors and random effects as in the alpha diversity model.

Finally, we modeled the association between the relative abundance of each microbial taxon (phylum, family, genus) and log-transformed neopterin index using LMMs, expecting that higher pathobiont abundance (e.g., *Helicobacter*, *Clostridium difficile*, *Escherichia-Shigella*) would predict higher neopterin indices in the following month (Fig. S5). For each microbial taxon, we included the relative abundance of the taxa (i.e., the number of reads per taxa and per sample divided by the total reads) as predictor of neopterin index, as well as the same control covariates and random effects as in the alpha and diversity models. We adjusted for multiple testing using the Benjamini–Hochberg correction.

All LMMs were run using the lmer function of the “lme4” package^50^ in R version 4.1.0^51^. All continuous predictors, excluding age, were scaled to facilitate model convergence. The significance of the fixed factors was tested using the “lmerTest” package^52^.

### Ethical approval

All data collection in Ethiopia was conducted with permission from the Ethiopian Wildlife and Conservation Authority, followed all laws and guidelines in Ethiopia, and was approved by the Institutional Animal Care and Use Committees at Arizona State University (20-1754 R) and Stony Brook University (773805_TR002). This research conformed to the American Society of Primatologists/International Primatological Society Code of Best Practices for Field Primatology and the ARRIVE guidelines.

## Results

### Technical validation

Neopterin (Kendall’s τ = 0.81, *p* = 0.01), creatinine (Kendall’s τ = 0.90, *p* = 0.003), and the neopterin index (Kendall’s τ = 0.71, *p* = 0.031) measurements derived from frozen aliquots were correlated with those derived from the same samples dried on filter paper (Fig. S2). Thus, dried samples closely reflect measurements drawn directly from urine. Indexed urinary neopterin and urinary creatinine (expressed in ng/mg) significantly decreased after the first four months of storage on filter paper (79.0 ± 13.2% percent recovery; Wilcoxon matched pairs test, v = 28, *p* = 0.016), but did not differ between 4 and 8 months of storage (Wilcoxon matched pairs test, v = 21, *p* = 0.297). However, sample measurements were still correlated across time points (0 vs. 4 vs. 8 months) for each measurement of interest (i.e., urinary neopterin, creatinine, and the neopterin index). Most critically for our study, neopterin indices measured shortly after sample collection were correlated with those assayed after 8 months of storage on filter paper (Kendall’s τ = 0.71, *p* = 0.031). Thus, degradation between zero and four months appears to have been generally consistent across samples and did not affect the rank order of samples. However, given that some samples were assayed more than 8 months after sample collection in the field, we included time to assay as a covariate in all applicable models.

### Demographic, environmental, and technical predictors of urinary neopterin

**Figure 1:**
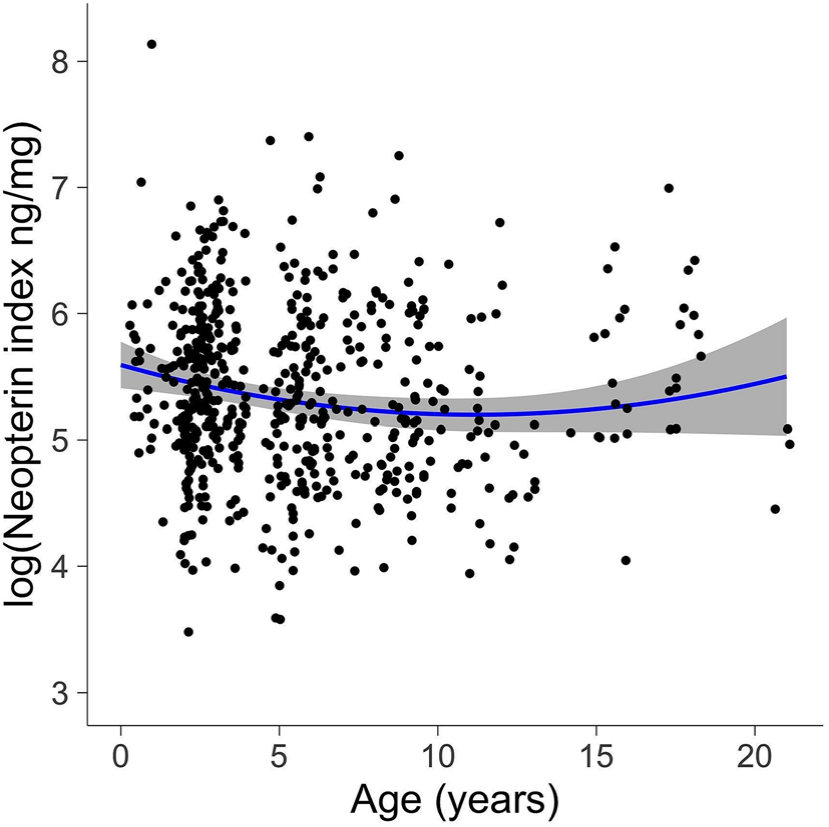
Log-transformed creatinine-corrected neopterin concentrations (neopterin:creatinine ng/mg) peak at early and older ages. The regression line and 95% confidence interval were generated with the R package ‘ggplot2’ for visualization; see Table 2 for estimates accounting for covariates and random effects. The marginal R^2^ (proportion of variance explained by the fixed effects) was 0.092, the conditional R^2^ (proportion of variance explained by the whole model) was 0.413, and the proportions of random effects variance explained by individual ID and batches were 0.086 and 0.267, respectively.

Across gelada samples, neopterin indices varied from 32.5 to 3413.5 ng/mg. Age and neopterin index had a U-shaped relationship in which very young and very old individuals showed the highest relative concentrations (Fig. 1), while neither rainfall nor minimum temperature was associated with variation in neopterin index (Table 2). Longer storage time and later time of day at collection were both positively associated with neopterin index (Table 2).

**Table 2:** Age, storage time, and collection time are associated with variation in urinary neopterin levels (ng/mg urinary creatinine, log-transformed) in geladas in the Simien Mountains National Park, Ethiopia.

Fixed effect	β	Std. Error	t-value	*p* value
Age	− 1.50	0.76	− 1.97	0.05*
Age^2^	1.98	0.73	2.71	< 0.01*
Sex (male)	− 0.03	0.07	− 0.44	0.66
Storage time (days)	0.15	0.07	2.20	0.04*
Time of day	0.08	0.02	3.09	< 0.01*
Cumulative rainfall (prev. 90 days)	0.07	0.06	1.21	0.24
Average min. temperature (prev. 30 days)	0.02	0.05	0.54	0.60

Asterisks indicate statistical significance (*p* ≤ 0.05).

### Larval tapeworm infection and neopterin

**Figure 2:**
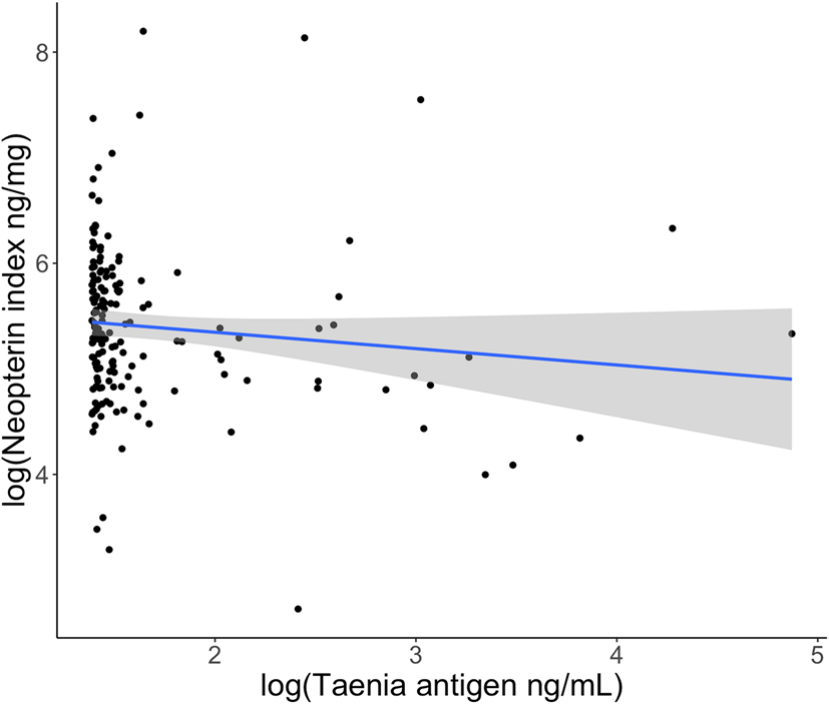
No association between log-transformed and creatinine-corrected *Taenia* antigen values and log-transformed neopterin index (neopterin:creatinine ng/mL). A small constant (4) was added to *Taenia* antigen concentrations to scale all concentrations to zero. The regression line and 95% confidence interval were generated with the R package ‘ggplot2’ for visualization; see Table 3 for estimates accounting for covariates and random effects. The marginal R^2^ (proportion of variance explained by the fixed effects) was 0.045, the conditional R^2^ (proportion of variance explained by the whole model) was 0.552, and the proportions of random effects variance explained by individual ID and batches were 0.411 and 0.130, respectively.

We found no association between neopterin index and *T. serialis* infection (Fig. 2; Table 3). Thirty samples from 19 individuals in our dataset had *Taenia* antigen concentrations above the indicative of infection (2.04 ng/mL; Fig. S3). Fourteen of 26 individuals (53.9%) from the GCCA population and five of 60 individuals (8.3%) from the SMNP population tested operationally positive (i.e., above 2.04 ng/mL) for *Taenia*. Three of 60 SMNP individuals (5%) and four of 26 GCCA individuals (15.4%) had cysts at the time of sampling or developed a cyst within one year of sample collection.

**Table 3:** No association between *Taenia* antigen concentration (ng/mL; corrected for creatinine, log-transformed) and urinary neopterin (ng/mg urinary creatinine, log-transformed) across both gelada populations.

Fixed effect	β	Std. Error	t-value	*p* value
Age	− 1.34	1.02	− 1.32	0.19
Age^2^	1.01	0.93	1.09	0.28
Time of day	0.07	0.06	1.23	0.22
Storage time (days)	0.12	0.12	1.02	0.33
*Taenia* antigen (corrected)	0.00	0.01	− 0.07	0.95
Sampling site	0.19	0.35	0.56	0.59

### Gastrointestinal helminth parasites and neopterin

**Figure 3:**
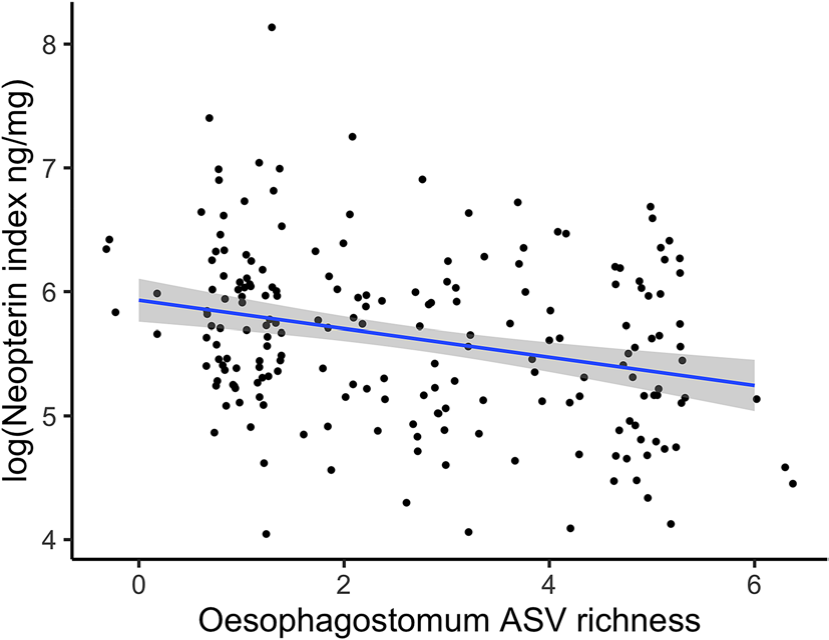
Higher richness of *Oesophagostomum* spp. helminth parasite amplicon sequence variants (ASVs) is associated with lower urinary neopterin index in geladas (neopterin:creatinine ng/mL). Neopterin samples were matched to all fecal parasite samples from the year prior to urine sample collection. Points are jittered for visualization. The regression line and 95% confidence interval were generated with the R package ‘ggplot2’ for visualization; see Table 4 for estimates accounting for covariates and random effects. The marginal R^2^ was 0.074, the conditional R^2^ was 0.392, and the proportions of random effects variance explained by individual ID and batches were 0.061 and 0.282, respectively.

We found a significant negative relationship between neopterin index and the richness of *Oesophagostomum* spp. ASVs (Fig. 3; Table 4), such that higher neopterin levels were associated with lower *Oesophagostomum* ASV richness over the year prior to neopterin sample collection. No other predictors were significant, including the richness of *Trichostrongylus* ASVs (Fig. S4). Across this subset of data, 100% of samples were positive for either *Trichostrongylus* spp. (97.9%) or *Oesophagostomum* spp. (96.4%), and 94.3% were positive for both.

**Table 4:** Higher richness of *Oesophagostomum* spp. helminth parasite amplicon sequence variants (ASVs) is significantly associated with lower urinary neopterin levels (ng/mg urinary creatinine, log-transformed) in geladas in the Simien Mountains National Park population.

Fixed effect	β	Std. Error	t-value	*p* value
Age	− 1.03	0.87	− 1.19	0.24
Age^2^	1.21	0.77	1.57	0.13
Time of day	0.06	0.09	0.61	0.55
Storage time (days)	0.04	0.05	0.83	0.41
Oesophagostomum ASV richness	− 0.08	0.03	− 2.45	0.02*
Trichostrongylus ASV richness	0	0.03	− 0.14	0.89

Asterisks indicate statistical significance (*p* < 0.05).

### Gastrointestinal microbiome composition and neopterin

**Figure 4:**
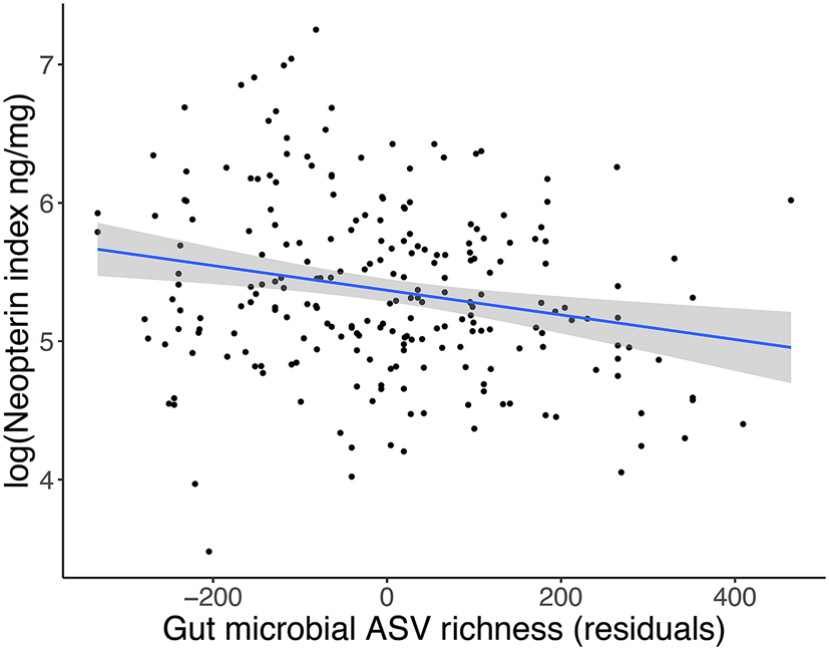
Higher microbial diversity (ASV richness) is associated with lower urinary neopterin index (neopterin:creatinine ng/mL) in geladas. Neopterin samples were matched to all gut microbiome samples from the month prior to urine sample collection. The regression line and 95% confidence interval were generated with the R package ‘ggplot2’ for visualization; see Table 5 for estimates accounting for covariates and random effects. The marginal R^2^ (proportion of variance explained by the fixed effects) was 0.146, the conditional R^2^ (proportion of variance explained by the whole model) was 0.368, and the proportions of random effects variance explained by individual ID and batches were 0.033 and 0.065, respectively.

Lower microbial alpha diversity was significantly associated with higher neopterin indices, and this pattern was consistent for all three metrics of alpha diversity: individuals with lower microbial ASV richness, evenness, and phylogenetic diversity had higher neopterin indices in the following month (Fig. 4; Table 5; Fig. S7; Table S1). None of the microbial community features of beta diversity (i.e., the first five PCs axes of a PCoA) significantly predicted neopterin indices (Fig. S8, Table S2), with the exception of PC5 based on Bray–Curtis dissimilarity (Fig. S8). We found no significant association between the relative abundance of phylum, family, or genus and neopterin indices after adjusting for multiple testing (Fig. S7; Table S3).

**Table 5:** Higher microbial alpha diversity (observed ASV richness) significantly predicts lower urinary neopterin (ng/mg urinary creatinine, log-transformed) in geladas of the Simien Mountains National Park population.

Fixed effect	β	Std. Error	t-value	*p* value
Age	− 0.38	0.68	− 0.56	0.58
Age^2^	0.71	0.66	1.09	0.28
Time of day	0.11	0.04	2.76	0.01*
Time to assay (days)	0.15	0.07	2.17	0.05*
Microbial ASV richness	− 0.10	0.04	− 2.57	0.01*

Asterisks indicate statistical significance (*p* < 0.05) following Benjamini–Hochberg correction for multiple testing.

## Discussion

Our results demonstrate that urinary neopterin captures variation in innate immunity related to age, gastrointestinal helminth infection, and gut microbiome alpha diversity in a wild primate. The youngest and oldest individuals in our dataset exhibited the highest neopterin levels, suggesting that urinary neopterin captures inflammation related both to the development of the immune system and to senescence. Individuals with a higher number of *Oesophagostomum* spp. amplicon sequence variants over the year prior to neopterin sampling had lower urinary neopterin, consistent with the expected anti-inflammatory phenotype induced by gastrointestinal parasites, while individuals with lower microbiome alpha diversity had higher urinary neopterin. Neopterin was not associated with larval tapeworm infection or environmental variables but was positively associated with time of sample collection and sample storage time. Altogether, these results suggest that urinary neopterin is a useful measure of innate immunity for certain metrics of health and disease throughout the lifespan in wildlife.

### Demographic, environmental, and technical predictors of urinary neopterin

The U-shaped association between age and neopterin in geladas recapitulates the pattern found in humans^23^, free-ranging mandrills^19^, and capuchins^24^, and is consistent with the patterns found in other studies that sampled from only one end of the age distribution^14,16–18^. High neopterin levels in infancy likely reflect the immune system’s first encounters with pathogens and parasites during the development of both innate and adaptive immune responses^53–55^, and high neopterin levels in old age likely arise from increased production of pro-inflammatory cytokines (including IFN-y, which stimulates neopterin-producing pathways) during senescence^53^. While an alternative explanation for this pattern is that individuals with the ability to produce high levels of neopterin are more likely to survive than others^56^, this is unlikely based on the demonstrated effects of inflammation on mortality^57^. Urinary neopterin can thus be a useful tool for the study of wildlife health and disease by facilitating the noninvasive study of early-age immune development and old-age immunosenescence in wild animals.

Both time of sample collection and sample storage time were positively associated with neopterin levels, pointing to the importance of their consideration in studies that implement filter paper-based sampling. In humans, neopterin and other immune components tend to increase in production overnight, with maximum measurements taken in the early morning^58–60^. In captive animals, no diurnal variation has been observed^15^, while our results point to yet an additional pattern: higher neopterin at later collection times. This may be because we are often unable to collect the first urine sample of the day as geladas sleep on and spend the first minutes of their mornings on steep cliffs. While we collected daily urine from 8:00am-4:30 pm, this points to the importance of collecting urine samples at similar times every day, particularly for accurate within-individual comparisons. Unlike neopterin in frozen liquid samples, which is stable at -20 °C for up to 24 months^14^, neopterin on dried filter paper samples stored at room temperature appears to degrade over the first four months. Despite these storage-related effects, the relative ranks of samples remained stable during our storage experiments. Although our actual sample storage time (up to two and a half years) exceeded the duration of our storage experiments (up to eight months) due to unavoidable and extenuating circumstances, most expected biological patterns were supported. Collectively, these results suggest that changes over time will not preclude comparisons but that storage time should be considered, and that the storage effects of filter paper may add noise but do not obscure the biological effects. While the effects of storage time on filter paper samples may potentially be mitigated by storing filter paper samples in a freezer^15^, this would undercut the benefits of filter paper collection at remote field sites without consistent access to electricity. Studies that implement neopterin in other species should, in addition to establishing parallelism and accuracy, evaluate the effects of these technical variables during validations and should consider assessing the relationship between age and neopterin as a metric of validation.

### Larval tapeworm infection and neopterin

As a product of white blood cells stimulated with a pro-inflammatory cytokine, neopterin is expected to reflect the activated cellular immune response in the face of pathogens that elicit inflammatory immune responses. We expected neopterin levels in geladas to be positively associated with *T. serialis* infection because taeniid larvae develop intramuscularly and are initially met with an eosinophil-based immune response^37,61^. Our lack of observed effect may be tied to the progression of the immune response over the course of *T. serialis* infection. Geladas become infected with *T. serialis* after incidentally ingesting infectious eggs shed in the feces of a carnivore infected with the adult stage of the parasite, after which asexually budding larvae develop in the gelada musculature, viscera, or soma^62^. These asexually budding infections can occur all over the body and often present as protuberant and highly conspicuous masses in geladas^39^. However, infections can also develop internally^1^, making it difficult to estimate the stage of infection.

In lab studies of closely related taeniids, larval establishment is met immediately with a primarily pro-inflammatory Th1 immune response that is replaced over time with a more regulatory and anti-inflammatory Th2 immune response^63,64^. One potential explanation for the lack of association between *T. serialis* infection and neopterin in our study is thus that our sampling approach captures infections during both Th1-dominant and Th2-dominant periods, effectively precluding our ability to parse this relationship. Because our *T. serialis* infection positivity threshold is based on individuals with cysts, we are likely to have a detection bias towards established, chronic infections that may not recruit the innate immune response at all. Alternatively, neopterin may not be a robust measure of the inflammatory immune response to intramuscular tapeworms at any stage of infection, just as it is not a useful marker of superficial injuries that are expected to recruit strong white blood cell responses^9,19^.

### Gastrointestinal parasites and neopterin

As anticipated based on the regulatory and anti-inflammatory immune phenotype induced by infection with gastrointestinal helminth parasites^41,65,66^, higher richness of unique sequences belonging to the gastrointestinal helminth genus *Oesophagostomum* over the year prior to urinary neopterin sample collection was associated with lower neopterin in geladas. Gastrointestinal helminths generally elicit Th2 polarized immune responses and initiate cascades of anti-inflammatory effector mechanisms, including the antibody-based immune response and regulatory T cell production, that facilitate their long-term establishment in hosts^33,41,67,68^. The Th2-polarized immune response blocks the expansion of the Th1 response, which includes the production of Th1 cells and their associated cytokines^69^. Principal among the Th1 cytokines suppressed by the Th2-polarized response is IFN-y—the primary catalyst of neopterin production by monocytes and macrophages^28^. The presence of an effect of ASVs belonging to *Oesophagostomum*, and not for *Trichostrongylus*, may reflect differences in the immune responses to each parasite and demands further investigation. Altogether, these results suggest that increased chronic richness of particular gastrointestinal parasite sequences down-regulates the pro-inflammatory immune response in geladas by suppressing Th1 effector mechanisms.

The anti-inflammatory effects of gastrointestinal helminth parasites may only be reflected in lower neopterin levels when measured longitudinally. The study on Barbary macaques^18^ assessed the effect of antihelminthic treatment on urinary neopterin levels over a ten-week period (six weeks prior to treatment and four weeks following treatment) and found no discernible effect of presence/absence metrics of helminth parasitism on neopterin. By contrast, we assessed the effect of helminth ASV richness over the year prior to neopterin sampling. This difference in effect was despite the two species exhibiting equally high rates of strongyle-type infections, with 100% prevalence of strongyle infections (*Oesophagostomum* spp., *Trichostrongylus* spp.) in geladas and 98% prevalence of strongyle infections (likely *Oesophagostomum* spp.) in macaques, with the addition of *Capillaria* and *Trichuris* sp. in the macaques. As the authors of the macaque study suggest, a single antihelminthic dose may not fundamentally alter the immunological phenotype induced by chronic helminth infection in wild animals. In addition, amplicon sequence variant richness or parasite load (i.e., egg count) may be more powerful metrics of gastrointestinal parasite community structure and associated immunological changes than presence/absence metrics.

### Gastrointestinal microbiome composition and neopterin

Despite the direct involvement of the gut microbiome in the production and activity of innate immune cells^70,71^, few studies have examined the relationship between gut microbiome composition and biomarkers of innate immunity^72–74^. Here, we found that geladas with low gut microbiome diversity exhibited higher neopterin levels over the following month. However, the lack of relationship between neopterin and abundances of specific microbial taxa or overall microbiota composition (i.e., beta diversity) suggest that higher neopterin indices are not linked to broad alterations of the microbial community or to specific microbiota profiles in geladas but rather to a depletion in microbial richness.

The association between lower microbiome diversity and higher neopterin levels in geladas may reflect the immunological consequences of microbiome disruption. The gut microbiome is a key regulator of immune homeostasis and participates in the polarization of the immune response^75–77^. In humans and laboratory animals, depletion of gut microbial diversity has been associated with immune dysregulation and chronic inflammation^78–82^ and higher susceptibility to infection^83,84^. Low microbial diversity in geladas may, for example, lead to a reduction in the production of anti-inflammatory microbial metabolites at the mucosal barrier (e.g., short-chain fatty acid such as butyrate) and allow for the proliferation of pro-inflammatory immune cells or otherwise catalyze an elevated pro-inflammatory response^74,85,86^ that would result in higher neopterin levels following low microbial diversity. Alternatively, the relationship between microbial diversity and neopterin in geladas could be the product of a complex three-way interaction between the immune system, the microbiome, and parasites, as gut microbes and helminths interact in overlapping ecological niches and the outcome of this interaction can influence the host immunological phenotype^87–90^.

## Conclusions

Altogether, our results demonstrate that urinary neopterin can be used for the non-invasive assessment of wildlife health and disease by measuring innate immune activity related to age, gastrointestinal helminth parasitism, and microbiome composition. We found that (1) very young and very old individuals had the highest neopterin levels, a pattern which may only be discernible if a study design includes adequate sampling across demographic categories, (2) very fine measures of chronic gastrointestinal helminth parasitism were associated with downregulated neopterin levels, and (3) lower microbiome diversity was associated with higher neopterin levels. In concert with other research that has shown the value of urinary or serum neopterin in predicting deaths from viral infections or reflecting viremia, our results suggest that urinary neopterin is an appropriate metric for assessing certain types of diseases (i.e., viruses, gastrointestinal helminths, microbiome dysbiosis) but not others (i.e., larval tapeworm infection). As anthropogenic activity continues to fragment habitats and alter the immunological and physiological phenotypes of wild animals, urinary neopterin will be a powerful tool for non-invasive assessment of health and disease in wildlife.

## Supplementary Information

Supplementary Information 1.

## Data Availability

Data and code are available on GitHub (https://github.com/GeladaResearchProject/Schneider_Crease_et_al_neopterin1).
